# Non‐chlorophyllous and crypto‐chlorophyllous fern spores differ in their mobilisation of fatty acids during priming

**DOI:** 10.1111/ppl.13848

**Published:** 2023-01-24

**Authors:** Luis V. Pedrero‐López, César M. Flores‐Ortiz, Blanca Pérez‐García, Rocío Cruz‐Ortega, Klaus Mehltreter, María E. Sánchez‐Coronado, Luis Barbo Hernández‐Portilla, Gastón Contreras‐Jiménez, Alma Orozco‐Segovia

**Affiliations:** ^1^ Instituto de Ecología Universidad Nacional Autónoma de México Ciudad de México Mexico; ^2^ Posgrado en Ciencias Biológicas Universidad Nacional Autónoma de México Ciudad de México Mexico; ^3^ Laboratorio de Fisiología Vegetal, UBIPRO, FES‐Iztacala Universidad Nacional Autónoma de México Tlalnepantla Mexico; ^4^ Laboratorio Nacional de Salud, FES‐Iztacala Universidad Nacional Autónoma de México Tlalnepantla Mexico; ^5^ Área de Botánica Estructural y Sistemática Vegetal, Depto. de Biología Universidad Autónoma Metropolitana‐Iztapalapa Ciudad de México Mexico; ^6^ Instituto de Ecología A. C. Carretera antigua a Coatepec Veracruz Mexico

## Abstract

During fern spore germination, lipid hydrolysis primarily provides the energy to activate their metabolism. In this research, fatty acids (linoleic, oleic, palmitic and stearic) were quantified in the spores exposed or not to priming (hydration–dehydration treatments). Five fern species were investigated, two from xerophilous shrubland and three from a cloud forest. We hypothesised that during the priming hydration phase, the fatty acids profile would change in concentration, depending on the spore type (non‐chlorophyllous and crypto‐chlorophyllous). The fatty acid concentration was determined by gas chromatograph–mass spectrometer. Chlorophyll in spores was vizualised by epifluorescence microscopy and quantified by high‐resolution liquid chromatography with a DAD‐UV/Vis detector. Considering all five species and all the treatments, the oleic acid was the most catabolised. After priming, we identified two patterns in the fatty acid metabolism: (1) in non‐chlorophyllous species, oleic, palmitic, and linoleic acids were catabolised during imbibition and (2) in crypto‐chlorophyllous species, these fatty acids increased in concentration. These patterns suggest that crypto‐chlorophyllous spores with homoiochlorophylly (chlorophyll retained after drying) might not require the assembly of new photosynthetic apparatus during dark imbibition. Thus, these spores might require less energy from pre‐existing lipids and less fatty acids as ‘building blocks’ for cell membranes than non‐chlorophyllous spores, which require de novo synthesis and structuring of the photosynthetic apparatus.

## 1 INTRODUCTION

Ferns have two independent life cycle phases: the gametophyte (haploid) and the sporophyte (diploid). The germination of spores (gametophyte), is like seed (sporophyte) germination in angiosperms, represents the beginning of their life cycle (Dyer, 1994; Sharpe et al., 2010). Both fern spores (unicellular) and angiosperm seeds (pluricellular) are resistant structures that preserve genetic information, disperse and preserve it in space and time (Hoekstra, 2002). Germination of seeds and fern spores begins with water uptake, which initiates the activation of their metabolisms, such as the mobilisation of energetic stored reserves (lipids, proteins, and starch), cell division and growth, and finally, germination becomes evident by the protrusion of the radicle in seeds and of the primary rhizoid or prothallial initial cell in fern spores (Raghavan, 1989: Bewley, 1997).

Despite the differences in their life cycle and structure, fern spores and seeds have functional similarities (Ballesteros et al., 2017). In both propagules, lipid content provides high energy to activate the metabolism during germination. Lipids also are the main components for repairing old and synthesising new cell membrane systems, such as organelles and primary cell walls and maintaining the structural integrity of membranes and their functions (Bewley, 1997; Lüttge, 2011). Consequently, during germination, mainly the fatty acids decrease as they are easily assimilated (Gemmrich, 1977a; Naguib, 2019; Robinson et al., 1973). In contrast, the fatty acids are present in large quantities in dry spores, where they are the energy reserves for cellular metabolism. Fatty acids are divided into two main groups: unsaturated fatty acids (linoleic and oleic), used for their energetic metabolism, and the saturated fatty acids (palmitic and stearic) that generate new unsaturated acids or used in the structure of their cell membranes. The spore also contains phospholipids in very small amounts; their function is to maintain the cellular structure and the cell membrane permeability (Gemmrich, 1977a; Robinson et al., 1973; Seilheimer, 1978).

In general, fern spores germinate 4–9 days after hydration. The energy resources of spores are obtained from the mother plant during their development in the sporangium. The lipid reserves in the spores are found in the cytoplasm as lipid globules or mixed with other components as proteins and pigments (Raghavan, 1989; Templeman et al., 1987). In fern spores, the lipid content varies from 4% in the genus *Ceratopteris thalictroides* (L.) Brongn., to 79% *Drynaria meyeniana* (Schott) Christenh. (formerly *Polypodium meyenianum* (Schott) Hook.) (Gemmrich, 1977a).

Functionally, three main types of spores have been described: non‐chlorophyllous, chlorophyllous and crypto‐chlorophyllous. The presence of chlorophyll determines their metabolic activity: chlorophyllous spores show short viability and fast germination, while non‐chlorophyllous spores have long viability, relative slow germination; these facts have consequences in gametophyte early establishment (Lloyd & Klekowski Jr, 1970). Although chlorophyll is present in the chlorophyllous and in the crypto‐chlorophyllous spores, it is observable only in the chlorophyllous (green spores); chlorophyll being covered by the perispore pigments in the crypto‐chlorophyllous spores, it is not visible to the naked eye or under optical or electronic microscopes (Sundue et al., 2011).

The hydration and activation of angiosperm seed metabolism is a triphasic process (Bewley, 1997); this occurs analogously in fern spores (Raghavan, 1989). During phase I, water diffuses into the spore to initiate its metabolism. The mobilisation of the reserves (proteins, lipids and carbohydrates) begins, and the mRNA is repaired and mobilised to repair cell components. During phase II, protein synthesis and reparation of cell membranes and mitochondria begin. In seeds and spores, carbohydrates are the first component to be metabolised followed by lipids. In spores, phase III is characterised by the protrusion of the first rhizoidal cell (Bewley, 1997; Raghavan, 1989). The events occurring in the three seed imbibition phases proposed by Bewley (1997) and the processes occurring during imbibition in spores (Raghavan, 1989) are homologous.

Priming treatments have been designed based on the stage of germination during seed and fern spore imbibition. These consist of one or more cycles of hydration–dehydration, where each cycle ends in a dehydration process to avoid radicle or rhizoid protrusion. All the biochemical and molecular changes occurring during imbibition phases I (fast water uptake) and II (imperceptible water uptake) remain even after storage in the dehydrated spore (Pedrero‐López et al., 2021). During the spore hydration periods, metabolic advances occur. When propagules are re‐hydrated, metabolism is re‐started and germination occurs fast and synchronously (Bray, 1995; Gamboa‐deBuen et al., 2006; Pedrero‐López et al., 2019). Invigoration during germination of primed fern spores suggests that a similar metabolic process occurs in both seeds and fern spores during priming. There are several priming treatments that regulate water uptake rate, among others: imbibition in water (hydropriming, HP) and imbibition in a matrix, such as soil (matrix priming, MP) (González‐Zertuche et al., 2001; Orozco‐Segovia et al., 2014; Pedrero‐López et al., 2019). These processes emulate the environmental conditions experienced in the soil by the small fern spores (40–60 μm) under natural conditions prior to spore germination (Pedrero‐López et al., 2019, 2021). Because priming treatments in fern spores generate a functional response that favours the invigoration of fern spores during germination (Pedrero‐López et al., 2019, 2021), we hypothesised that, as part of the metabolic and biochemical changes occurring during hydration, fern spore fatty acids will change in the amount and/or composition compared to non‐primed spores. Lipids will be mobilised and/or catabolised because they are required during imbibition phases I and II to reinitiate spores metabolism. Non‐chlorophyllous and crypto‐chlorophyllous spores might exhibit a different lipid metabolism.

## 2 MATERIAL AND METHODS

### 2.1 Study area and plant material

Spores from five species of ferns were collected in 2012–2013 from two localities: *Pleopeltis thyssanolepis* (A. Braun ex Klotzsch) E.G. Andrews & Windham (formerly *Polypodium thyssanolepis* Klotzsch, rupicolous and epiphyte), and *Pellaea ovata* (Desv.) Weath. (rupicolous) at the ecological reserve Parque Ecológico de la Ciudad de México (PECM) located in the municipality of Tlalpan, Mexico City, Mexico; and *Alsophila firma* (Baker) D.S. Conant (arborescent), *Sphaeropteris horrida* (Liebm.) R.M. Tryon (arborescent) and *Amauropelta rudis* (Kunze) Pic. Serm. (formerly *Thelypteris rudis* (Kunze) Proctor, terrestrial) at the municipality of Tlatlauquitepec, Puebla, Mexico. The studied species were selected based on their ability to produce a high number of spores. In the PECM, the vegetation is xerophilous shrubland growing on a lava field; the Köppen climate is Cb′(w2)(w) (temperate by elevation, with a long sub‐humid fresh summer) with a mean annual temperature of 14°C and a mean annual precipitation of 880 mm (González‐Hidalgo et al., 2002). In Tlatlauquitepec, the vegetation consists of a cloud forest under a Köppen climate (A)Ca(fm)(e) (subtropical with rains throughout the year) with a mean annual temperature of 20.2°C and a mean annual precipitation of 1243 mm (CONAGUA, 2022; Instituto de Geografia, 2004). All spores were obtained from fertile leaves of 5–10 individuals. Pinnae with closed sporangia were placed inside manila envelopes, labelled, and sealed. To dehydrate and liberate spores from the sporangia, the envelopes were placed on a flat surface in the laboratory in the dark (21.6 ± 1.8°C, relative humidity = 38.7%). Spores were sieved with a phytoplankton net with a mesh of 0.74 μm. Finally, they were placed in glass vials at 5°C. Vouchers of each species were deposited in the herbarium of the Universidad Autónoma Metropolitana‐Iztapalapa (UAMIZ, LVPL 3–7).

### 2.2 Priming treatments applied to fern spores

During the same year of spore collection, diverse treatments were applied to fern spores: hydropriming (HP), matrix priming (MP) and non‐primed spores as control (C). Treatments were applied to each species in triplicate. Each replicate contained 100 mg of spores weighed in an analytical balance (OHAUS GA200, OHAUS Corp.) and placed inside phytoplankton net bags (25 μm in opening) (3 treatments × 5 species × 3 replications). For HP, net bags were immersed in tap water, while for MP they were buried 3 cm deep in pots (100 ml) filled with soil from the collection areas and watered to field capacity. Based on the lag time for germination of all studied species, hydration was applied for 8 days in the dark. After the hydration periods, bags were recovered from the hydration substrate and dehydrated for 2 days on a table surface inside the darkroom to avoid germination induction. Finally, spores were kept in closed glass vials until they were used for lipid extraction and quantification. Fatty acids extraction was carried out in a room at 4°C.

### 2.3 Extraction of fatty acids

Two years after collection, spores were treated and then fatty acids were extracted from control and treated spores of *S*. *horrida* (C, HP and MP treatments), *Alsophila firma* (C, HP and MP), *Pleopeltis thyssanolepis* (C, HP and MP), *Pellaea ovata* (C and HP) and *Amauropelta rudis* (C and MP). The number of treatments differed among species because some samples were insufficient after spore trituration to perform all the fatty acids analyses. For each species, spores of the three replicates were mixed and an aliquot of 100 mg of spores was triturated at 4°C in a porcelain mortar containing glass powder. To the triturated sample, we added 1 ml of chloroform:methanol (2:1) solution. The supernatant was transferred to microcentrifuge tube vials that were shaken in a Vortex for 5 min to recuperate the organic layer. The supernatant was washed with 200 μl of NaCl at 9% and centrifuged at 18,000 × *g* at 10°C. To extract the fatty acids, we mixed 500 μl of the organic phase and 100 μl of the internal standard Heptadecanoic acid 1 mg ml^−1^ was used for fatty acid quantification and evaporated in a microcentrifuge tube vial (Brinkmann Instruments Inc., Centrifuge 5415C). Subsequently, 500 μl of BF_3_ (boron trifluoride at 12% in methanol) was added. The tube was closed and sealed with sealing tape and placed in boiling water for 20 min. Finally, 500 μl of hexane and 1000 μl of distilled water were added to the tube, the tube was manually shaken, and the organic phase was recovered for the analysis of fatty acids, which was performed in triplicate.

For the analysis of the methyl esters of fatty acids, we followed the method of Cabrera‐Santos et al. (2021). To identify the fatty acids, a gas chromatograph was used (GC; Agilent Technologies 6850), coupled with a mass spectrometer (MS; Agilent Technologies 5975C VL MSD). For the GC system, a DB‐1 (dimethylpolysiloxane) capillary column was used (30 m length × 0.32 mm i.d., 5.00 μm film thickness, part number: 123‐1035 E, Agilent Technologies 6850). The oven temperature was programmed as follows: from 100°C; ramp 1: to 250°C with 5°C min^−1^. The injector temperature was 200°C in split mode. Helium was used as a gasifier fatty acids carrier at a linear flow velocity of 35 cm s^−1^ or 1.4 ml min^−1^. The transfer line of the mass detector was kept at 250°C, the mass detector parameters ranged from 20 to 400 m/z, positive polarity, the ionisation energy of 70 eV, and a temperature of 200°C, with an injection volume of 2 μl. The results of the mass spectra were compared with the NIST/EPA/NIH Mass Spectral Library 2020 version.

### 2.4 Determination of chlorophyll in spores

Crypto‐chlorophyllous spores has been reported in tree ferns (i.e. Cyatheaceae; Sundue et al., 2011; Tseng et al., 2017); however, the presence of crypto‐chlorophyllous spores was assessed in all the five species independent of the plant family. Ten years after spores collection, in viable spores of each species, images were acquired using a microscope with epifluorescence and laser microdissection functions (Arcturus XT—Nikon eclipse Ti, Applied Biosystems). The microdissection function was used at a wavelength of 349 nm to generate a cutting laser beam, of about 45 mW power, able to cut spores. To identify both chlorophylls *a* and *b* in the microdissected spores, the pieces were immediately observed under the microscope under a light source ranging from 330 to 750 nm wavelengths (halogen lamp) through a 20× objective lens, a set of filters was used to delimitate the specific excitation and emission spectra for both chlorophylls *a* and *b*. For chlorophyll *b*, we used an excitation filter with a bandwidth of 475/15 nm, and for chlorophylls *a*, we used an excitation filter with a bandwidth of 375/15 nm, using a mercury lamp. Additionally, a dichromatic mirror cutoff of 395 nm long pass was used, the emission for both chlorophylls *a* and *b* of the spores was filtered by an emission filter with a bandwidth of 635/35 nm and collected by a CCD colour camera 1024 × 768 pixels (The Imaging Source). Extraction of photosynthetic pigments with methanol (including the accessory pigments) was performed 2 years after spore collection by high‐resolution liquid chromatography (HPLC), using the reverse phase (Olives et al., 2006), with the following conditions: Allsphere ODS‐1 column (250 × 4.6 mm) of 5 μm particle size, constant flow of 2 ml min^−1^, the mobile phase consisted of a gradient containing acetonitrile:methanol:Tris–HCl buffer, 0.1 M pH 8 (75:12:4) as phase A, while phase B consisted of methanol:hexane (80:20). The gradient started with 100% A and held for 5 min, then changed gradually to 100% B during 2.5 min, and held for 7.5 min with 100% B, the total run time was 15 min. A diode array detector was used in a range of 380–800 nm and with detection at 663 nm. Finally, due to the lack of sufficient spores, an analytical quantification with an ultraviolet/visible light spectrophotometer of chlorophylls *a* and *b* was done only for *S. horrida* (UV/Vis spectrophotometer, Perkin Elmer Lambda S2, Perkin Elmer Corp.). The content of chlorophyll *a*, chlorophyll *b* and the total were determined using the models of Coombs et al. (1985).

### 2.5 Data analyses

In control spores, the effect of the species, fatty acids type (sources of variation, categoric variables) and their interaction on the concentration (dependent variable, numeric) of the total of the fatty acids evaluated in this research were tested with a two‐way analysis of variance (anova).

For each species, the effect of priming treatments and fatty acid type (sources of variation, categoric variables) on fatty acid concentrations (dependent variable, numeric) were tested with a two‐way anova. In all cases, the normality of the data was tested with Shapiro–Wilk test (*p* > 0.05) and Tuckey's tests as post hoc test and homocedasticity was tested with Levene's tests (*p* > 0.05). Statistical analyses were performed with Statgraphics Centurion 15 v 15.2.05 software.

To estimate, in each species, the magnitude of the change in the concentrations of each fatty acids type, as an effect of the priming treatments, we also calculated the relative changes in the amount of each one of the fatty acids due to the treatments, in respect to the concentration in control spores (concentration of fatty acids in primed spores/control).

## 3 RESULTS

### 3.1 Fatty acid content

In control spores, the study found significant effects of the species, fatty acids type and their interaction (*F*_(4,59)_ = 99.67, *p* < 0.001; *F*_(3,59)_ = 212.79, *p* < 0.001 and *F*_(12,59)_ = 70.60, *p* < 0.001, respectively) on the total concentration of the four types of fatty acids evaluated in this research. Both *Pellaea ovata* and *Pleopeltis thyssanolepis* showed the highest concentration of fatty acids, mostly due to their significantly high concentration of oleic acid. *Amauropelta rudis* showed an intermediate trend with significant higher values for linoleic and oleic acids than *Alsophila firma* and *Sphaeropteris horrida*, which had the significantly lowest concentration of all fatty acid types (Table 1).

**TABLE 1:** Total concentration of the fatty acids included in this study (palmitic, linoleic, oleic and stearic acids), in control spores of five fern species collected in the ecological reserve Parque Ecológico de la Ciudad de México (*) or in the municipality of Tlatlauquitepec, Puebla, Mexico (ɸ)

Species	Total concentration of fatty acids (mg g^−1^)
*Alsophila firma* ɸ	10.66 ± 3.57c
*Sphaeropteris horrida* ɸ	12.01 ± 12.48c
*Amauropelta rudis* ɸ	69.91 ± 41.91b
*Pellaea ovata* *	131.07 ± 161.95a
*Pleopeltis thyssanolepis* *	121.49 ± 194.66a

*Note*: Mean values ± standard deviations. Different letters indicate significant differences (*p* < 0.05).

Significant differences were mainly found in oleic acid concentrations. In any case, stearic acid had the lowest concentrations (2.15–18.82 mg g^−1^) and had no significant changes in any species or treatments.

**FIGURE 1:**
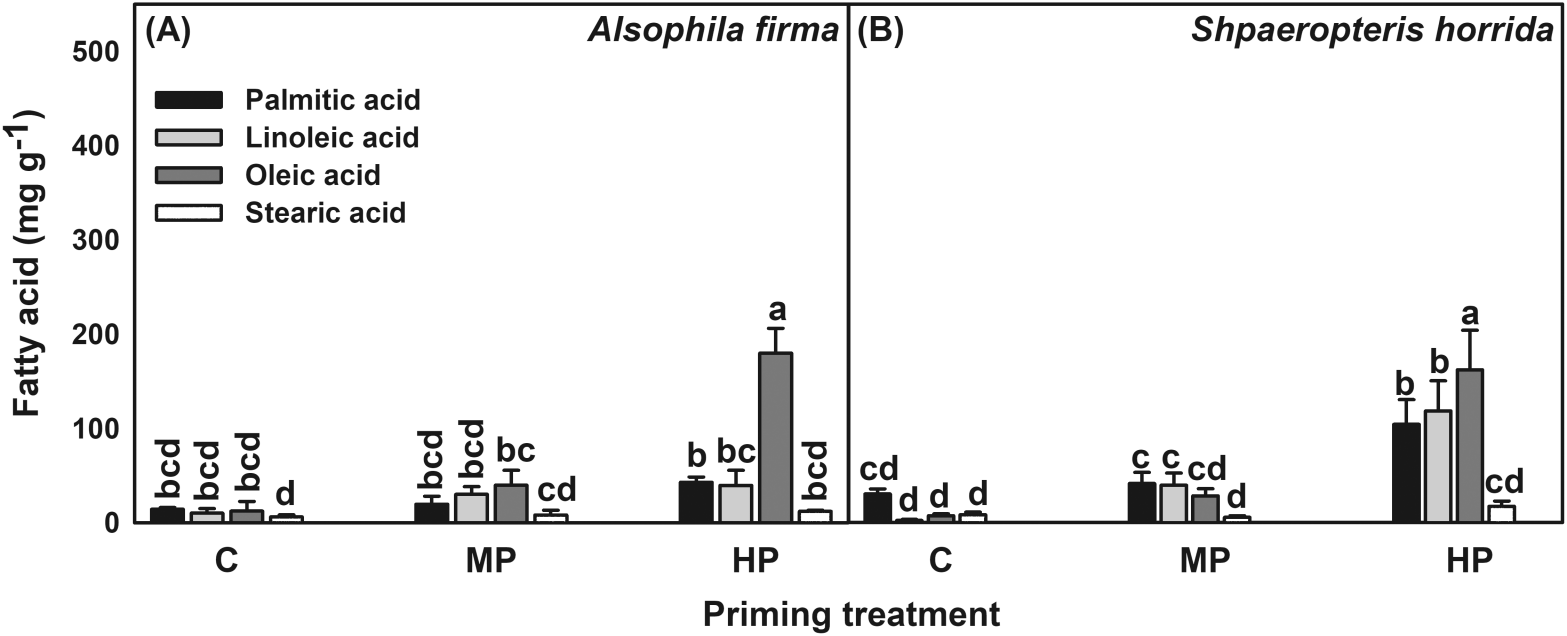
Effect of priming on fatty acid content on spores of (A) *Alsophila firma* and (B) *Sphaeropteris horrida*. Control (C), hydropriming (HP), matrix priming (MP). Data shown means ± sd, lowercase letters on bars indicate significant differences according Tukey test (*p* < 0.05, *n* = 3)

In *Alsophila firma*, treatments, fatty acid type and their interactions induced significant differences in fatty acid concentrations (*F*_(2,35)_ = 87.35, *p* < 0.001; *F*_(3,35)_ = 62.86, *p* < 0.001 and *F*_(6,35)_ = 38.8, *p* < 0.001, respectively). Only oleic acid had an increase inconcentration after HP (14.65 times; 180.06 mg g^−1^) compared to control (12.29 mg g^−1^; Figure 1A).

In *S. horrida*, treatments, fatty acid type and their interaction induced significant differences in fatty acids concentrations (*F*_(2,35)_ = 81.5, *p* < 0.001; *F*_(3,35)_ = 17.3, *p* < 0.001 and *F*_(6,35)_ = 10.34, *p* = 0.001, respectively). After HP, the concentration in oleic (161.9 mg g^−1^), linoleic (118.24 mg g^−1^) and palmitic (104.09 mg g^−1^) acids increased in relation to non‐primed spores (7.18, 2.16, and 30.28 mg g^−1^, respectively). When compared to controls, the increase in palmitic, linoleic and oleic acids in HP was 3.43, 54.74 and 22.54 times, respectively. In MP, only linoleic acid (39.66 mg g^−1^) increased 18.36 times in relation to control (2.16 mg g^−1^; Figure 1B).

**FIGURE 2:**
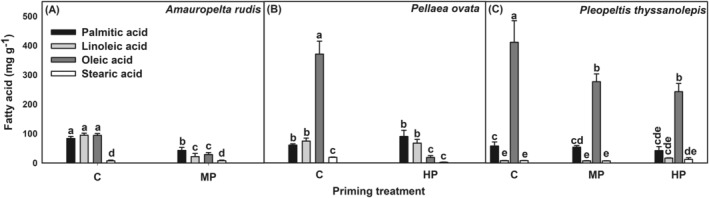
Effect of priming on fatty acid content on spores of (A) *Amauropelta rudis*, (B) *Pellaea ovata* and (C) *Pleopeltis thyssanolepis*. Control (C), hydropriming (HP), matrix priming (MP). Data shown means ± sd, lowercase letters on bars indicate significant differences according Tukey test (*p* < 0.05, *n* = 3)

For *Amauropelta rudis*, fatty acid determinations were done only for control and MP. In this species, priming, fatty acids type and their interaction were significant (*F*_(1,23)_ = 231.2, *p* < 0.001; *F*_(3,23)_ = 83.11, *p* < 0.001 and *F*_(3,23)_ = 31.6, *p* < 0.001, respectively). MP induced a decrease in the concentration of palmitic, linoleic, and oleic acids (42.92, 21.91 and 28.74 mg g^−1^, respectively) compared to their concentrations in control (83.43, 94.60 and 94.04 mg g^−1^, respectively) by 1.94 (palmitic acid), 4.31 (linoleic acid) and 3.27 (oleic acid) times (Figure 2A).

For *P. ovata*, fatty acid determinations were done only for C and HP. Significant differences were induced for treatments, fatty acid type and their interaction (*F*_(1,23)_ = 133.7; P < 0.001, *F*_(3,23)_ = 105.88; P < 0.001 and *F*_(3,23)_ = 141.5; P < 0.001). Only oleic acid reduced its concentration after HP (18.43 mg g^−1^) by 20.11 times compared to control (370.63 mg g^−1^, Figure 2B).

For *Pleopeltis thyssanolepis*, treatments, fatty acid types and their interaction induced significant decreases in the fatty acid concentrations (*F*_(2,35)_ = 10.23, *p* < 0.001, *F*_(3,35)_ = 307.23, *p* < 0.001 and *F*_(6,35)_ = 9.69, *p* < 0.001, respectively). In control, oleic acid concentration was 411.35 mg g^−1^ and decreased 1.48 times in MP (277.07 mg g^−1^) and 1.69 times in HP (243.05 mg g^−1^) (Figure 2C).

### 3.2 Determination of chlorophyll in spores

**FIGURE 3:**
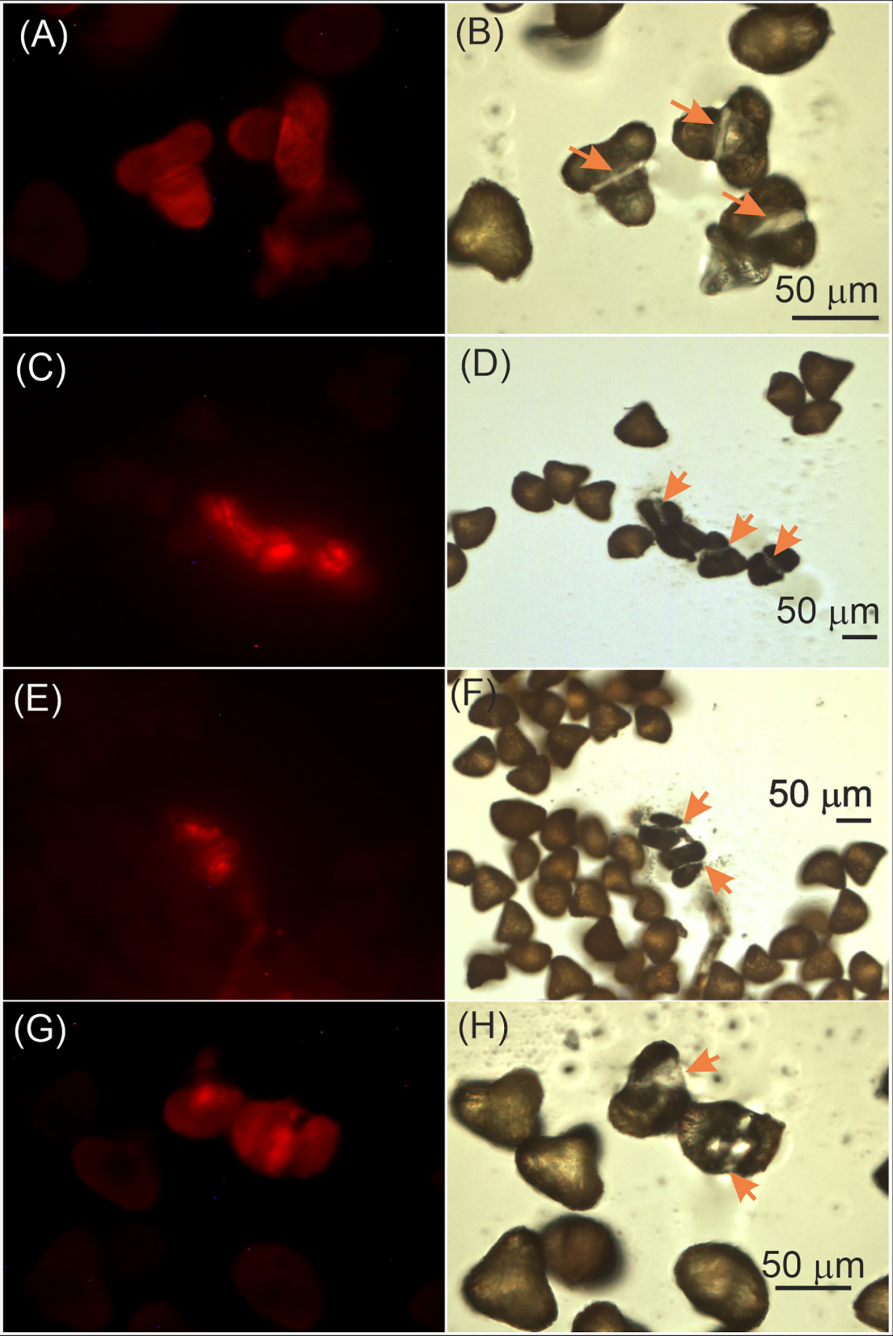
Photographic comparison of spores of: *Sphaeropteris horrida* (A–D) and *Alsophila firma* (E–H). Spores were photographed with: epifluorescence microscopy with UV light (A,E), epifluorescence microscopy with blue light (C,G) or white light microscopy (B,D,F,H)

**FIGURE 4:**
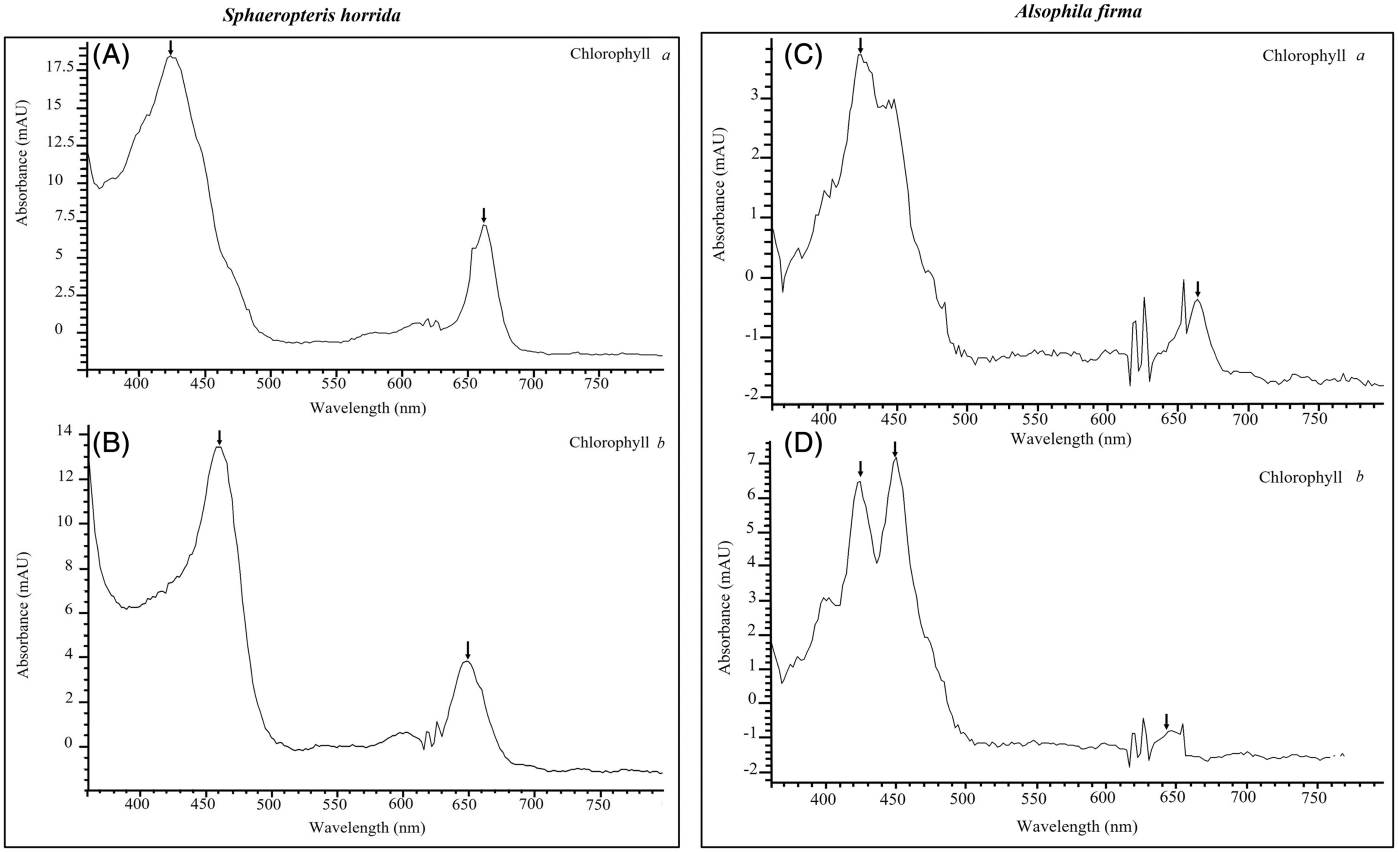
Absorption spectrum of chlorophyll *a* (A,C) and chlorophyll *b* (B,D), analysed by HPLC. Fern species are indicated on the top of the figure

In the micro‐dissected spores, we observed their emission for *S. horrida* and *Alsophila firma* (Figure 2). A second verification test of chlorophylls was carried out with a high‐resolution liquid chromatography (HPLC). Both species presented emission spectra corresponding to chlorophylls *a* and *b* (Figures 3 and 4). *Sphaeropteris horrida* contained 7.975 μg g^−1^ of total chlorophyll, 4.67 μg g^−1^ of chlorophyll *a* and 2.72 μg g^−1^ of chlorophyll *b*. *Pellaea ovata*, *Pleopeltis thyssanolepis* and *Amauropelta rudis* did not present fluorescence associated to the chlorophyll presence and did not present emission spectra corresponding to chlorophylls *a* and *b*.

## 4 DISCUSSION

The priming treatments induced different changes in the concentrations of the fatty acids, depending on the species, which suggests differential metabolism of fatty acids during priming, as it occurs in seeds (Espanany et al., 2016; Naguib, 2019). Based on fluorescence microscopy, HPLC and UV/Vis spectroscopy, *Pleopeltis thyssanolepis*, *Pellaea ovata* and *Amauropelta rudis* have non‐chlorophyllous spores, while *S. horrida* and *Alsophila firma* have crypto‐chlorophyllous spores. During priming, spores of both types stayed hydrated 8 days in darkness, thus absorbed water (imbibition phase I) and remained in imbibition phase II. As a result of priming, we observed two patterns in the fatty acids concentrations: one for non‐chlorophyllous species and another one for the two crypto‐chlorophyllous species.

Several of the processes occurring in spores, mainly during the activation of germination (sensu stricto; Bradford, 1995), might explain the contrasting patterns observed in fatty acids concentration after priming. In the non‐chlorophyllous species, oleic acid was consumed. In *Amauropelta rudis*, palmitic acid and linoleic acid were also catabolised. The lipid reduction might be the result of the catabolic activities occurring in darkness during the priming treatments, as occurs in *Daucus carota* L. seeds (Zhao et al., 2021), or in fern spores during imbibition (Sato & Furuya, 1984). In general, monounsaturated fatty acids have a greater capacity to be metabolised during priming (Walters et al., 2005).

In contrast, in the primed crypto‐chlorophyllous species, fatty acids' concentration increased. Oleic acid increased in *Alsophila firma*, and linoleic, oleic and palmitic acids increased their concentration in *S. horrida* after HP.

An explanation for these two patterns might be that spores of *S. horrida* and *Alsophila firma* have an initially low fatty acids content and might be using other type of reserves, such as globulin proteins (DeMaggio & Stetler, 1985). Thus, in these species, mobilisation and high synthesis de novo of fatty acids would occur during imbibition (phases I and II) to cover the subsequent gametophyte development and growth (Robinson et al., 1973). However, lipids, not proteins, are the main reserve in fern spores (Gemmrich, 1977b). Contrastingly, spores of non‐chlorophyllous *Pleopeltis thyssanolepis*, *Pellaea ovata* and *Amauropelta rudis* have an initial high fatty acids content and their catabolism was mainly observed in primed spores compared to control. It is difficult to find research articles that could support this idea since fatty acids content has been determined in relatively few fern spores species. Only Gemmrich (1977a) includes the concentration of 8 fatty acids in spores of 17 species, 16 non‐chlorophyllous species and one chlorophyllous.

The increase in fatty acids after priming also suggests that this response might be linked to biochemical metabolic differences occurring during imbibition phases I and II. This might be related to the initial chlorophyll content of the spores. Some species of tree ferns (Cyatheaceae), such as *Sphaeropteris lepifera* (Hook.) R.M.Tryon (Cyatheaceae), are crypto‐chlorophyllous (Tseng et al., 2017), which makes an important functional difference compared to non‐chlorophyllous fern spores. In our study, we showed that *S. horrida* and *Alsophila firma* were crypto‐chlorophyllous.

In the germination activation phase (imbibition phase II), the requirements needed for the subsequent gametophyte or embryo development and growth ought to be initiated or covered (Bradford, 1995; Raghavan, 1989; Robinson et al., 1973). In the homoiochlorophylly found in desiccation‐tolerant plant species, chlorophyll is retained after tissues or cells dehydration and reconstituted during rehydration (Shivaraj et al., 2021; Tuba et al., 1994; Tuba et al., 1998), as occurs in the homoiochlorophyllous sporophyte of *Pleopeltis polypodioides* (L.) E.G. Andrews & Windham (John & Hasenstein, 2018, 2020; López‐Pozo et al., 2019). This might also occur in crypto‐chlorophyllous and chlorophyllous spores; thus, chlorophyll reconstitution and repair of organelles might occur simultaneously during Phase II of imbibition of the priming treatments. Contrarily, in the imbibed state, the non‐chlorophyllous fern spores require cell membrane repair, organelles assemblage and differentiation, and the synthesis of chlorophyll and other photosynthetic pigments, to complete photomorphogenesis during subsequent exposure to light and later produce functional chloroplasts (Gemmrich, 1980). In this research, the hydration phase of priming treatments occurred in darkness. For different taxa, including ferns, it has been reported that complete or partial development of photosynthetic apparatus occurs in darkness (Armstrong, 1998; Xue et al., 2017). The complete photosynthetic morphogenetic process might require lipids as energy source and/or as ‘building blocks’ of membrane lipids (sensu de Carvalho & Caramujo, 2018) from the pre‐existing fatty acids, as it has been reported during chloroplast morphogenesis in darkness for the algae *Chromochloris zofingiensis* (Dönz) Fucíková & Lewis (Zhang et al., 2020).

Other processes linked to hydration–dehydration–rehydration treatments (as in priming treatments) might also explain the increase in fatty acids content observed after dehydration at the end of the priming treatments in *Alsophila firma* and *S. horrida*. In the homoiochlorophyllous *P. polypodioides*, an increase in fatty acids content after dehydration has been considered as (1) an adaptation aimed to prevent lipid peroxidation caused by the increase of reactive oxygen species (ROS) in the components of the pre‐existing photosynthetic apparatus, or (2) an adaptation to desiccation stress where fatty acids play a role of stress‐responsive metabolites in cell membranes, depending on temperature (John & Hasenstein, 2018, 2020). Additionally, the unsaturated fatty acids increase also protect the photosynthetic apparatus from damage (Gombos et al., 1994; John & Hasenstein, 2020).

Nonetheless, increases in fatty acids concentration coming from the mobilisation of other lipids and synthesis of de novo fatty acids ought to be assessed to understand the increases in fatty acids concentrations found in these two tree ferns during priming. Because we identified *S. horrida* and *Alsophila firma* as species with crypto‐chlorophyllous spores, it seems relevant to assess this spore trait in other fern species as well.

During priming treatments, it might occur catabolism, mobilisation or de novo synthesis of fatty acids that might increase the fern spore vigour (i.e. germination synchronisation, reduction in lag time and/or increase in germination rate) during germination as previously reported for primed fern spores of the species included in this research (Pedrero‐López et al., 2019, 2021). Several authors have reported changes in the concentrations of fatty acids during fern spore germination (DeMaggio & Stetler, 1985; Gemmrich, 1977a; Raghavan, 1989; Seilheimer, 1978), but these changes are not clearly related to the biochemical changes occurring in the dark before germination, as in *Daucus carota* seeds during the imbibition (Zhao et al., 2021).

## 5 CONCLUSION

In fern spores, the application of priming treatments caused changes in the concentrations of fatty acids, mainly oleic acid, which is an easily assimilable chemical component in fern spores, necessary for the metabolic activation of the spores and prepare them to germinate and to establish in their environment.

Changes in the fatty acids content in the studied species showed that these reserves are mobilised and/or catabolised during imbibition phases I and II to reinitiate the metabolism of spores. Fatty acids, in fern spores are the same as those stored in seeds, although, in fern spores, the lipids metabolism occurs on a smaller scale inside a single cell compartment. The fatty acids mobilisation differed between non‐chlorophyllous and crypto‐chlorophyllous spores as a result of priming. Non‐chlorophyllous species catabolised oleic, palmitic, and linoleic acids, while crypto‐chlorophyllous species increased their concentration, suggesting that crypto‐chlorophyllous spores with homoiochlorophyll might not require fatty acids for the assembly of the photosynthetic apparatus during dark imbibition. Alternatively, fatty acids might prevent lipid peroxidation or be stress‐responsive metabolites, as suggested by previous reports for sporophyte of *P. polypodioides*. However, it is necessary to assess de novo synthesis of fatty acids during dark imbibition at different temperatures in crypto‐chlorophyllous spores.

Although matrix priming simulated the permanence of spores in the soil, hydropriming caused a more pronounced changes in fatty acids concentrations than matrix priming. This suggests that water availability in the soil (saturated soils vs. unsaturated soils) might be relevant for germination and the establishment of gametophytes in the field.

## AUTHOR CONTRIBUTIONS

Luis V. Pedrero‐López: conceptualisation, investigation, methodology, data collection and analysis, writing review and editing; César M. Flores‐Ortiz: supervision, writing, review and editing, resources, validation; Blanca Pérez‐García, Rocío Cruz‐Ortega, Klaus Mehltreter: supervision, writing, review and editing; María E. Sánchez‐Coronado: formal analysis, writing—review and editing; Luis Barbo Hernández‐Portilla: data processing and methodology; Gastón Contreras‐Jiménez: data collection and processing, final—draft; Alma Orozco‐Segovia: conceptualisation, investigation, supervision, funding acquisition, methodology, resources, validation, writing—original draft, writing—review and editing.

## CONFLICT OF INTEREST

The authors declare that they have no known competing financial interests or personal relationships that could have appeared to influence the work reported in this paper.

## Data Availability

All data are available in the manuscript. Statistical analysis are available if they are required.
